# *Plasmodium knowlesi* – Clinical Isolate Genome Sequencing to Inform Translational Same-Species Model System for Severe Malaria

**DOI:** 10.3389/fcimb.2021.607686

**Published:** 2021-03-02

**Authors:** Damilola R. Oresegun, Cyrus Daneshvar, Janet Cox-Singh

**Affiliations:** Division of Infection, School of Medicine, University of St Andrews, St Andrews, United Kingdom

**Keywords:** *Plasmodium knowlesi*, MinION, parasite virulence, severe malaria, translational model system

## Abstract

Malaria is responsible for unacceptably high morbidity and mortality, especially in Sub-Saharan African Nations. Malaria is caused by member species’ of the genus *Plasmodium* and despite concerted and at times valiant efforts, the underlying pathophysiological processes leading to severe disease are poorly understood. Here we describe zoonotic malaria caused by *Plasmodium knowlesi* and the utility of this parasite as a model system for severe malaria. We present a method to generate long-read third-generation *Plasmodium* genome sequence data from archived clinical samples using the MinION platform. The method and technology are accessible, affordable and data is generated in real-time. We propose that by widely adopting this methodology important information on clinically relevant parasite diversity, including multiple gene family members, from geographically distinct study sites will emerge. Our goal, over time, is to exploit the duality of *P. knowlesi* as a well-used laboratory model and human pathogen to develop a representative translational model system for severe malaria that is informed by clinically relevant parasite diversity.

## Background

Malaria is a vector-borne disease that has impacted human health in tropical and sub-tropical regions since ancient time and continues to outwit human endeavors to control and eradicate. Malaria parasites, genus *Plasmodium*, have a highly complex lifecycle, intimately dependant on an invertebrate mosquito host for the diploid sexual stage of reproduction and equally dependant on specific vertebrate hosts for asexual replication and transmission. Lifecycle complexity, including adaptation to specific vertebrate hosts, invertebrate host restriction to particular Anopheline vector species with spatial and ecological niche requirement may augur unfavourably for *Plasmodium* spp. survival in a dynamic world. Yet, despite sustained efforts, human malaria persists to the extent that the altruistic World Health Organization (WHO) malaria eradication goal of the 1950s, was downgraded to country and at times species-specific elimination https://www.who.int/malaria/areas/elimination/en/. Even so, eradication is not a forgotten dream and may well be achievable within a new 30-year time-frame (Feachem et al., 2019).

The human host-adapted *Plasmodium* species; *Plasmodium falciparum*, *Plasmodium vivax*, *Plasmodium malariae*, and *Plasmodium ovale*, two sub-species (Sutherland et al., 2010) are responsible for most of the reported cases of malaria. *P. falciparum*, in particular, and *P. vivax* are responsible for the global health burden of disease. *P. falciparum* infections carry a high level of morbidity and mortality in adults and children. Severe malaria manifests variously, for example as severe malaria with coma, acute kidney injury and severe malarial anaemia (Plewes et al., 2018; White, 2018; World-Health-Organization, 2019). Understanding the underlying pathophysiology of severe malaria is thwarted by the absence of a translational model system. In practice, malaria elimination remains the most effective strategic method to reduce indigenous transmission of *P. falciparum* and/or *P. vivax* and consequently the impact of severe malaria. Malaria elimination is a long-term goal and in the meantime people will continue to be infected and succumb to severe malaria.

Malaria elimination status is awarded to each country by the WHO even though the country need not necessarily be malaria free. A case in point is Malaysia where indigenous human-host adapted *Plasmodium* species transmission is zero and malaria elimination status was expected to be awarded to Malaysia by the WHO in 2020 (Liew et al., 2018; Jiram et al., 2019; Noordin et al., 2020) https://www.who.int/malaria/areas/elimination/e2020/malaysia/en/. However, malaria - the disease, persists in Malaysia, particularly in the eastern states of Sabah and Sarawak where for the past 20 years *Plasmodium knowlesi*, a malaria parasite of macaque monkeys, has been regularly diagnosed in symptomatic patients in Sabah and Sarawak (Lee et al., 2009a; Barber et al., 2017; Cooper et al., 2020; Raja et al., 2020).

## *Plasmodium knowlesi* Malaria

As one millennium closed and a new one began, a substantial number of cases of *P. knowlesi* were identified in the human population in the Kapit division of Sarawak Malaysia Borneo (Singh et al., 2004). The entry of *P. knowlesi* into the human population became apparent as the number of cases of *P. falciparum* and *P. vivax* declined in response to robust control programmes. Up to that point *P. knowlesi*, a parasite morphologically similar to both *P. malariae* and the early trophozoites of *P. falciparum*, was misdiagnosed by routine microscopy (Lee et al., 2009b). Misdiagnosis as *P. falciparum* had little clinical consequence as both infections require urgent treatment and management. Misdiagnosis as the more benign *P. malariae* resulted in delayed treatment and the development of severe disease and preventable fatality (Cox-Singh et al., 2008).

There is no indication that the cases of *P. knowlesi* malaria are decreasing, 69% of the 16,500 reported cases of malaria in Malaysia between 2013 and 2017 were caused by *P. knowlesi* (Raja et al., 2020) (Hussin et al., 2020). In 2018, more than 4,000 cases of malaria were reported in Malaysia and with *P. falciparum* and *P. vivax* close to elimination, *P. knowlesi* accounted for most of those (World-Health-Organization, 2019; Chin et al., 2020).

*P. knowlesi* malaria is also widespread across South East Asia where the natural habitat supports sylvan transmission – areas where the specific Anopheline vectors of *P. knowlesi*, the natural macaque hosts and the parasites co-exist and where humans enter these habitats (Singh and Daneshvar, 2013; Shearer et al., 2016). Zoonotic malaria is unlikely to fill the void left by the removal of *P. falciparum* and *P. vivax*, however, communities living close to and individuals who enter the jungle transmission areas for work or leisure activities are at risk of this newly emergent potentially life threatening disease.

*P. knowlesi* malaria is associated with severe disease in 10 – 12% of cases with death in vulnerable and untreated individuals (Cox-Singh et al., 2008; Daneshvar et al., 2009; William et al., 2011; Rajahram et al., 2012; Grigg et al., 2018; Hussin et al., 2020). Although *P. knowlesi* infections are associated with hyperparasitaemia, severe malaria caused by *P. knowlesi* occurs across a wide spectrum of parasitaemia. Relatively low parasite counts, ≥15,000 parasites/µl carry a high risk of severe disease (Willmann et al., 2012; Cooper et al., 2020). Severe *P. knowlesi* malaria is characterised by one or more of the WHO criteria for severe malaria including; anaemia, acute kidney injury, acute and late-onset respiratory distress, hypotension, jaundice, and metabolic acidosis (Cox-Singh et al., 2008; Daneshvar et al., 2009; Cox-Singh et al., 2010; Barber et al., 2011; World-Health-Organization, 2013; Grigg et al., 2018). Indeed, until the discovery of zoonotic malaria caused by *P. knowlesi*, severe malaria was the preserve of *P. falciparum* and severe malaria guidelines written for *P. falciparum* infection. With few tools to study the pathways to severe malaria and the absence of a comparator disease, assigning clinical cause and effect in malaria was roadblocked.

**Figure 1:**
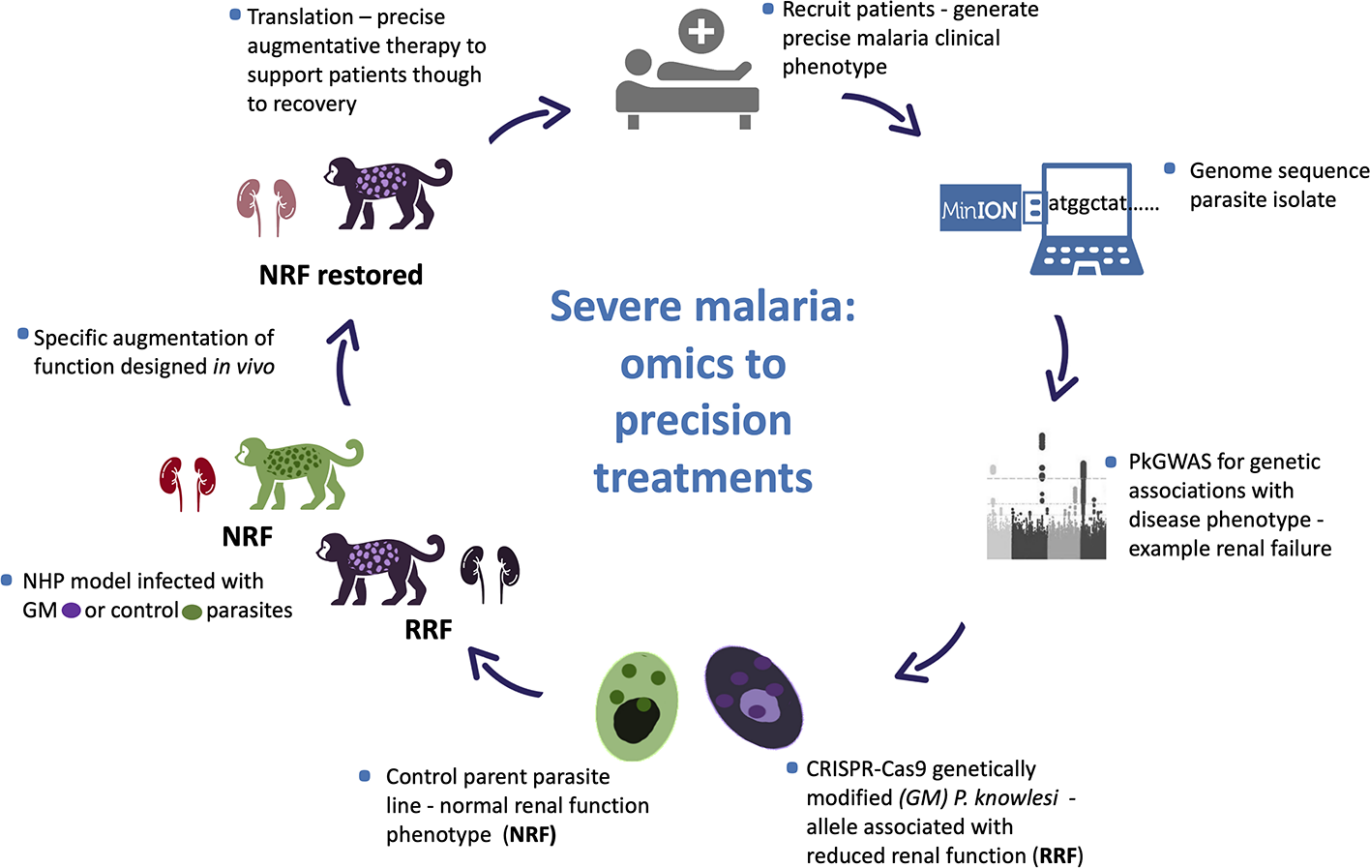
*Plasmodium knowlesi* - proposed translational and dynamic model system for severe malaria. Precise clinical phenotyping malaria patients coupled with parasite genome sequencing “bed-side”. Parasite genome wide association studies (pGWAS) with markers of disease – an example of renal impairment in severe *P. knowlesi* infection is used here. Associating alleles are superimposed onto an experimental parasite genetic background to produce genetically modified parasites (GM). GM parasites phenotyped *in vitro* and *in vivo* (non-human primate -NHP, model) for matched disease phenotype. NHP model used to develop strategies to specifically reduce the impact of the clinical phenotype for translation into therapies for patients with severe malaria.

Even so characterising and untangling the combined contribution of human host response and pathogen to disease presentation and outcome is inherently complex, in the literal sense. The human race survives and often thrives in a harsh microbial world (Numbers, 2011). Of the many microbes only a few are pathogenic, and even-then not uniformly so. Host innate immune function is key to infectivity with co-factors, including age, co-morbidity and co-evolution influencing disease outcomes. Such disease determinants are poorly defined, yet critical to understand, as witnessed in the ongoing Coronavirus pandemic (Mills et al., 2015; Loy et al., 2017; Mandl et al., 2018; Petersen et al., 2020). Human host diversity and response to infection, including response to infection with potentially virulent malaria parasites, is outside of the scope of this article. Rather we focus on determining clinically relevant *Plasmodium* spp. genetic diversity and propose a model system to test for association between parasite genetic diversity and clinical outcome (**Figure 1**).

It is also perhaps worth noting here that in general, disease phenotype precision remains a limiting factor in genome-wide associations studies (GWAS) and an area that lags behind available technologies (MacRae, 2019). No different is the study of parasite genetic characters associated with disease progression (pGWAS) in malaria where disease phenotype precision is currently lacking. No matter how sophisticated the model system, mathematical, *in silico*, *in vitro*, or *in vivo*, the outputs can only be as precise as the input data.

Modelling malaria is especially difficult because malaria parasites have co-evolved with their vertebrate hosts each exerting selective forces on the other in a dynamic dance for survival (Loy et al., 2018). That dynamic is complicated further because malaria parasites are eucaryotic with a relatively large genome, 20–40 mega bases organised into 14 chromosomes (Gardner et al., 2002; Carlton et al., 2008; Pain et al., 2008; Ansari et al., 2016). Designing experiments to identify the drivers of pathogenesis, of parasite virulence and disease cause and effect are challenging.

The advent of “omics” may better inform models for malaria through multiple data generation platforms; genomics, transcriptomics and proteomics (Pinheiro et al., 2015; Campino et al., 2018; Benavente et al., 2019; Lindner et al., 2019). Even with these tools all too often, information is extrapolated. Rodent models and experimental lines that are unable to capture clinically relevant parasite diversity are much better characterised for markers of parasite virulence than diverse contemporary clinical isolates (Plewes et al., 2018). The value of supporting sophisticated forward genetic screens on laboratory isolates with clinical isolate genotyping was demonstrated in a recent study on *P. falciparum* gene clusters involved in erythrocyte invasion (Campino et al., 2018). Invasion phenotypes generated from crossing two experimental lines and phenotype-associated deletions were compared with long-read sequence data available from a small number of clinical isolates where indels in the same large locus were identified supporting invasion pathway variation in nature. Ignoring the impact of natural pathogen diversity on disease progression and virulence creates an inexcusable vacuum when analysing data for parasite association with disease severity.

*P. knowlesi* is an adaptable and naturally diverse parasite. A genetic study on clinical isolates of *P. knowlesi* identified an association between certain haplotypes of a short polymorphic fragment (~885bp) of the *Plasmodium knowlesi normocyte binding protein (Pknbp)xa* on chromosome 14 and continuous data on markers of disease severity (Ahmed et al., 2014). In addition to disease association, the fragment was dimorphic, clinical isolates clustered into one of two distinct genotypes at that locus, begging the question how far the dimorphism extended across the *Pknbpxa* 9578bp gene and chromosome 14.

Harnessing the power of next-generation sequencing seemed the obvious choice to take this work forward within the caveat that the clinical isolates of *P. knowlesi* available to study were small volume (<1mL) frozen whole blood. Undeterred and as proof of concept, we developed a method to deplete human DNA and concentrate parasite DNA in the samples. We produced *P. knowlesi* genome sequences from six clinical isolates using massively parallel Illumina short-read sequencing platforms (Pinheiro et al., 2015). The move from genetics to genomics for clinical isolate characterization unlocked a wealth of information. Subsequent analyses found that the *Pknbpxa* dimorphism extended along the gene and chromosome 14. Indeed, SNPs associated with the dimorphism were found on all chromosomes and involved more than half of all genes in *P. knowlesi* parasites isolated from patients. The work demonstrated that *P. knowlesi* isolated from human infections in Sarawak, Malaysian Borneo are one of two distinct genotypes.

Both pieces of work unlocked hidden genome-wide characters in clinical isolates of *P. knowlesi*. The studies reinforced the idea that pathogen genome sequence data extracted from clinically well characterised infections provides a valuable resource for studies on the role of pathogen diversity in virulence and disease outcome.

## *P. knowlesi –* A Model for Malaria

*P. knowlesi* was first described in a long tailed macaque in the 1930s (Knowles and Gupta, 1932). Early work demonstrated that *P. knowlesi* was an adaptable parasite and experimental lines were developed and maintained in rhesus macaques, *Macaca mulatta*, to model for malaria. The *P. knowlesi* – rhesus macaque malaria model was used extensively for studies on malaria antigenic variation, vaccine development, parasite invasion, and biology, recently reviewed (Butcher and Mitchell, 2018; Galinski et al., 2018; Pasini et al., 2018). Traditionally *P. knowlesi* was not favoured as a model for disease, pathophysiology, mostly because the *P. knowlesi* in *Macaca mulatta* was particularly aggressive and not representative of human malaria caused by *P. falciparum*. A view supported in more recent work on cytokine responses in *M. mulatta* experimentally infected with *P. knowlesi* where a dampened response, and if anything, an anti-inflammatory response was observed in this model, a response that is uncharacteristic of human-host *Plasmodium* infections (Praba-Egge et al., 2002). *P. falciparum* malaria and indeed *P. knowlesi* clinical infections, are characterised by vigorous pro- and anti-inflammatory responses depending on age and endemicity (Cox-Singh et al., 2011; Farrington et al., 2017). Taken together there was little support for the utility of *P. knowlesi* in *M. mulatta* as an *in vivo* model for severe malaria. *P. knowlesi* in other experimental non-human primates (NHP’s) produces a disease more representative of human malaria and it is surprising that this opportunity to model severe malaria has not been taken forward (Langhorne and Cohen, 1979; Ozwara et al., 2003; Onditi et al., 2015). Unfortunately lack of support for using *P. knowlesi* to model for severe malaria is compounded by evolutionary distance. *P. knowlesi* and *P. falciparum* occupy distinct phylogenetic clades and phylogenetic distance is often used to argue against using *P. knowlesi* to model for *P. falciparum*. Evolutionary distance continues to be used to question the validity of comparing *P. knowlesi* with *P. falciparum* malaria, yet they are member-species of the same genus – by definition they are closely related. In practice, evolutionary distance often over-rides biological and comparable clinical characters and *P. knowlesi* is more often favourably viewed as a model for the phylogenetically closer yet phenotypically quite distinct *P. vivax* (Moon et al., 2013; Mohring et al., 2019; Verzier et al., 2019).

Neither *P. falciparum* nor *P. vivax* is permissive in intact experimental NHP hosts and to date, representative heterologous translational models for malaria are not available to interrogate pathways to pathology and to develop augmentative therapies. Consequently, the treatment and management of patients severely ill with malaria remain imprecise and generally supportive. We argue that clinical data collected from patients with naturally acquired severe *P. knowlesi* coupled with homologous laboratory adapted, well characterised and genetically adaptable experimental lines of *P. knowle*si can be exploited to discover the parasite drivers of severe malaria. Laboratory adapted lines of *P. knowlesi* are permissive in a range of NHP hosts, including olive baboons and common marmosets (Langhorne and Cohen, 1979; Ozwara et al., 2003; Onditi et al., 2015). Some of these *in vivo* models exhibit clinical characters representative of severe malaria caused by *P. falciparum* and, importantly, contemporary clinical descriptions of severe malaria caused by *P. knowlesi* (Cox-Singh et al., 2008; Daneshvar et al., 2009; Cox-Singh et al., 2010; Daneshvar et al., 2018). Notwithstanding NHP models are of ethical concern, expensive and valid only if the information obtained significantly advances knowledge, which often is not the case. Experimental lines of *P. knowlesi* even if modelled *in vivo* are effectively research silos lacking the power to inform clinical disease caused by genetically diverse contemporary wild-type zoonotic parasites (Ahmed et al., 2014; Assefa et al., 2015; Divis et al., 2015; Pinheiro et al., 2015).

Clinical descriptions of *P. knowlesi* malaria portray a spectrum of disease from uncomplicated – to severe and fatal infections and can be compared phenotypically with *P. falciparum* malaria (Cox-Singh et al., 2008; Daneshvar et al., 2009; Cox-Singh et al., 2010; Cox-Singh et al., 2011; Rajahram et al., 2012; Ahmed et al., 2014; Barber et al., 2018a; Barber et al., 2018b).

The duality of *P. knowlesi* as an adaptable experimental model and human pathogen offers a unique opportunity to develop a comprehensive representative translational model system for malaria informed by same-species clinical disease.

Two important advances enhance the utility of *P. knowlesi* as a model for disease. The first is the adaptation of an experimental line of *P. knowlesi* to *in vitro* growth in human erythrocytes (Moon et al., 2013). The second is transfection technology. *P. knowlesi* in macaque erythrocytes was already shown to be more amenable to transfection, meaning genetic modification, than experimental lines of *P. falciparum* (Kocken et al., 2002). The human erythrocyte adapted line is similarly receptive to transfection and indeed CRISPR-Cas9 targeted genetic modification technology, genome editing, has been developed for *P. knowlesi* (Moon et al., 2013; Mohring et al., 2019). These technologies together with genome sequence data, generated from clinical isolates, will facilitate the introduction of clinically relevant alleles of *P. knowlesi* into experimental lines for *in vitro* characterisation and the unique opportunity to take this work forward *in vivo* (Cox-Singh and Culleton, 2015).

A long journey to cause and effect harnessing omics, genetically modified parasites and comprehensive model systems to properly ascribe parasite virulence to malaria pathophysiology while possible is a long game, difficult, time-consuming and expensive. However, failure to make this effort is to perpetuate acceptance of clinical and therapeutic blind-spots, imprecise and generally supportive treatment and management for severe malaria, that perhaps is only acceptable if there is no alternative.

In the first instance, the ability to genome sequence *Plasmodium* species isolated from clinically well-characterised malaria patients will facilitate *Plasmodium* Genome-Wide Associations Studies (*p*GWAS) and identify virulence gene candidates. We show how short and long-read *Plasmodium* genome sequence data can be generated from fresh or archived frozen samples held in the many malaria research centres worldwide. *Plasmodium* genome sequence outputs over time and space will facilitate the construction of a substantial genetic reference resource, based on diverse wild-type parasites isolated from patients, that will inform model systems (Milner et al., 2012; Ahmed et al., 2014; Pinheiro et al., 2015; Auburn et al., 2018; Campino et al., 2018; Divis et al., 2018; Otto et al., 2018; Su et al., 2019; Siao et al., 2020). Until now genome sequence data generated from clinical samples was more feasible using Illumina massively parallel short-read sequencing.

**Figure 2:**
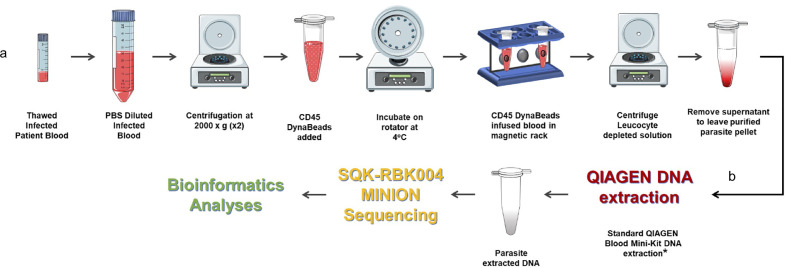
Protocol for processing low volume frozen whole blood samples from patients with malaria in preparation for third generation genome sequencing. **(A)** Human leucocyte depletion and **(B)** sequencing pipeline. *Human DNA was quantified using qPCR (Klaassen et al., 2003) and a standard curve derived from Human Genomic Control DNA (Applied Biosystems^®^, TaqMan^®^). In the absence of pure control parasite DNA, cycle threshold (*ct*) values from *P. knowlesi* qPCR (Divis et al., 2010) were normalised by volume and used to estimate parasite DNA enrichment following human DNA depletion. *Parts of the figure are conceptualised and adapted using Servier Medical Art, Servier: *https://smart.servier.com.

As highlighted in the *P. falciparum* invasion gene study described in an earlier section, prohibitively expensive and otherwise impractical long-read genome sequencing data from clinical isolates were required to validate study findings (Campino et al., 2018). We have already developed a method to extract *P. knowlesi* DNA from archived small volume clinical isolates suitable for Illumina short-read sequencing (Pinheiro et al., 2015). To overcome limitations of short-read genome sequencing that are problematic for multiple repeat regions and multiple gene families in *Plasmodium* spp., the method was further adapted for Oxford Nanopore MinION long-read sequencing, third-generation sequencing, that is accessible, affordable, mobile and suitable for low yield DNA samples (**Figure 2**). Briefly 200–400ul samples of archived whole blood from *P. knowlesi* patients was rapidly thawed and immediately diluted in 50mL cold PBS. The suspension was mixed gently before recovering parasites and any contaminating white blood cells (WBC’s) by centrifugation: 2,000 x g; 20 minutes; 4°C (pellet 1). The supernatant was transferred to a fresh tube and centrifugation repeated to maximise parasite recovery (pellet 2). The pellets were combined and resuspended in 1.2mL of cold PBS. Surviving host WBCs, a source of human DNA (hDNA) contamination, were removed using magnetic beads coated with antibodies to the ubiquitous WBC surface marker CD45 (Dynabeads™ CD45, Invitrogen™). Dynabeads were prepared as per manufacturers’ instruction and 100ul bead suspension added to the 1.2mL pellet suspension followed by incubation at 4°C with rotation for 30 minutes. WBC’s bound to the beads were removed by placing in a magnetic field for two minutes.

The human WBC depleted eluate was carefully removed and transferred to a fresh Eppendorf tube and centrifuged at 2,000 x *g* for 20 min to recover the parasite enriched pellet (PEP). The PEP was suspended in 200ul PBS for DNA extraction (QIAamp DNA Blood Mini Kit, QIAGEN). Recovered DNA was eluted in 150ul of Buffer AE. Percent hDNA depletion and Plasmodium DNA recovery were determined using quantitative PCR (qPCR) (Klaassen et al., 2003; Divis et al., 2010). *P. knowlesi* qPCR *ct* values negatively correlated with genome coverage (*p* = 0.0375). *P. knowlesi* DNA enriched samples (post hDNA depletion) from isolates with a starting parasitaemia of <40,000 per ul had low parasite DNA yield and sequence coverage.

Twenty-one (21) samples from 15 different patients, median parasitaemia 193,600 parasites/ul (IQR 127,875 – 321,750; min 20,656; max 794,063) with >90% hDNA depletion were taken forward to PCR-free rapid barcoding library preparation (Oxford Nanopore, SQK-RBK004). SQK-RBK004 library preparation is suitable for small yield DNA samples in the region of 400ng and includes a tagmentation step that generates read lengths normally distributed around a mean length of 4,500 kb. Of 21 library preparations 13 (62%) had >10x genome sequence coverage and six of these >30x coverage. Coverage of 100x was achieved especially when >1 sequencing library was prepared per isolate.

For the first time it is possible to generate long-read *Plasmodium* genome sequence data from small clinical samples from malaria patients. Samples that are archived or collected prospectively can be sequenced in a cost-effective and time-efficient manner anywhere. The importance of this capability is the opportunity to move forward from a necessary dependence on *Plasmodium* genome sequence generated from experimental lines to genome sequence generated from clinical samples with matched clinical data. The methodology we describe is particularly applicable to *P. knowlesi* infections that tend to be single genotype and reach relatively high parasitaemia. The methods can be applied to, albeit, relatively uncommon single genotype *P. falciparum* infections. Although multiple genotype infections present a limiting factor to the methods described here, the potential to generate valuable genome-wide information on even a small number of clinical isolates to inform studies on *P. falciparum* virulence should not be overlooked.

Subsequent *p*GWAS on long read sequence data from clinical isolates with matched high quality continuous clinical and laboratory data, relative to particular clinical manifestations of severe malaria, will help unravel the contribution of parasite diversity to virulence. In addition to accessibility and field application of low-cost real-time sequencing in-house, Nanopore MinION long-read sequencing can resolve important multiple gene family members, including the *P. knowlesi kirs* and *SICAvars* (Pain et al., 2008; Pinheiro et al., 2015; Lapp et al., 2018). These and other gene clusters encode surface antigens that are implicated in malaria parasite virulence and are difficult to sequence, formerly requiring expensive sequencing platforms equally prohibitive in cost and quantity of input DNA required (Campino et al., 2018).

Our particular interest is to use MinION sequence data from clinical isolates of *P. knowlesi* in *p*GWAS studies. We will analyse matched continuous clinical data predictive of precise characteristics of severe malaria to identify candidate alleles implicated in virulence to take forward in functional studies. CRISPR-Cas9 technology developed for *P. knowlesi* (Mohring et al., 2019) will facilitate locus-specific gene editing to superimpose clinically relevant alleles onto experimental lines and offer the opportunity for allele-specific phenotyping *in vitro*. Genetically modified lines with *in vitro* phenotypic characters that carry a high suspicion of involvement in parasite virulence and following exhaustive experimentation will be deemed suitable to take forward *in vivo* for clinical phenotyping and translational research (**Figure 1**).

Our immediate goal is to promote third-generation genome sequencing and capacity strengthening in bioinformatics for routine genetic studies on clinical malaria in endemic countries. The outputs will create a repository that captures diversity and information on multiple gene families hitherto outside the remit of all but large centres mostly working on model parasites. Our long-term vision is to develop a precise experimental model system for severe malaria pathophysiology informed by clinical infections and culminating in *in vivo* disease phenotyping and translational research. A model that, for the first time, will have the power to characterise parasite allele-specific cause and effect. A model system that exploits the utility of *P. knowlesi*, a laboratory model, and *P. knowlesi* that is responsible for naturally acquire human disease.

Not the end of the story or perfect by any standard but our sequencing capability represents a significant step forward towards creating the means to understand malaria pathophysiology and to inform the rational design and development of adjunctive therapies for patients with severe malaria.

## Data Availability Statement

The raw data supporting the conclusions of this article will be made available by the authors, without undue reservation.

## Ethics Statement

The studies involving human participants were reviewed and approved by University of St. Andrews. The patients/participants provided their written informed consent to participate in this study.

## Author Contributions

JC-S and CD conceived the work and wrote the manuscript. DRO did sample processing, sequencing, and bioinformatics. All authors contributed to the article and approved the submitted version.

## Funding

DRO is supported by the Wellcome Trust ISSF award 204821/Z/16/Z. Bioinformatics and computational biology analyses were supported by the University of St Andrews Bioinformatics Unit (AMD3BIOINF), funded by Wellcome Trust ISSF award 105621/Z/14/Z. The sample BioBank was compiled with informed consent (Medial Research Council, www.mrc.ac.uk, grant G0801971). Genome sequencing was supported by Tenovus Scotland (T16/03).

## Conflict of Interest

The authors declare that the research was conducted in the absence of any commercial or financial relationships that could be construed as a potential conflict of interest.
